# Crosstalk between G-quadruplex and ROS

**DOI:** 10.1038/s41419-023-05562-0

**Published:** 2023-01-18

**Authors:** Songjiang Wu, Ling Jiang, Li Lei, Chuhan Fu, Jinhua Huang, Yibo Hu, Yumeng Dong, Jing Chen, Qinghai Zeng

**Affiliations:** Department of Dermatology, Third Xiangya Hospital, Central South University, 138 Tongzipo Road, 410013 Changsha, Hunan PR China

**Keywords:** Chromatin structure, Senescence

## Abstract

The excessive production of reactive oxygen species (ROS) can lead to single nucleic acid base damage, DNA strand breakage, inter- and intra-strand cross-linking of nucleic acids, and protein-DNA cross-linking involved in the pathogenesis of cancer, neurodegenerative diseases, and aging. G-quadruplex (G4) is a stacked nucleic acid structure that is ubiquitous across regulatory regions of multiple genes. Abnormal formation and destruction of G4s due to multiple factors, including cations, helicases, transcription factors (TFs), G4-binding proteins, and epigenetic modifications, affect gene replication, transcription, translation, and epigenetic regulation. Due to the lower redox potential of G-rich sequences and unique structural characteristics, G4s are highly susceptible to oxidative damage. Additionally, the formation, stability, and biological regulatory role of G4s are affected by ROS. G4s are involved in regulating gene transcription, translation, and telomere length maintenance, and are therefore key players in age-related degeneration. Furthermore, G4s also mediate the antioxidant process by forming stress granules and activating Nrf2, which is suggestive of their involvement in developing ROS-related diseases. In this review, we have summarized the crosstalk between ROS and G4s, and the possible regulatory mechanisms through which G4s play roles in aging and age-related diseases.

## Facts

- ROS can lead to single nucleic acid base damage, DNA strand breakage, inter- and intra-strand cross-linking of nucleic acids and protein-DNA cross-linking.
- G4 is a four-stranded nucleic acid structure formed by guanine-rich DNA and RNA sequences with Hoogsteen hydrogen bonding.
- The structure and biological regulatory role of G4s can be affected by cations, helicases, transcription factors, G4-binding proteins, and epigenetic modifications.

## Open questions

- How do ROS affect the stability of G4 and G4-mediated biological regulation?
- What are the mechanisms through which G4s regulate the transcription and translation of ROS-related genes?
- Are G4s potential therapeutic targets in ROS-mediated aging and related diseases?

## Introduction

The imbalance between the production and clearance of reactive oxygen species (ROS) triggers extensive damage to cellular components such as nucleic acids, proteins, and lipids [1]. Low levels of ROS act as signaling molecules that regulate proliferation, angiogenesis, and metastasis [2]. However, high levels of ROS can lead to apoptosis and/or necrosis by inducing oxidative damage to nucleic acids with single base damage, DNA strand breakage, inter- and intra-strand cross-linking of nucleic acids, or protein-DNA cross-linking [3–5]. The DNA bases, especially guanine (G), are highly susceptible to oxidation due to their low redox potential. The oxidized base 8-oxoguanine (OG) is recognized and excised by 8-oxoguanine DNA glycosylase 1 (OGG1) following activation of the DNA damage response (DDR) pathway [6, 7]. A disproportionate level of oxidative stress and an aberrant DDR machinery can lead to accumulated damaged DNA and trigger apoptotic pathways [8], which is the pathological basis of aging and aging-related diseases [9, 10].

G-quadruplex (G4) is a four-stranded nucleic acid structure formed by guanine-rich DNA and RNA sequences with Hoogsteen hydrogen bonding [11, 12]. Computational predictions indicate that G4 motifs are prevalent and enriched in human gene promoters compared to the rest of the genome, which strongly suggest a role in gene regulation [13, 14]. G4s are likely involved in multiple biological processes ranging from telomere lengthening to DNA replication, transcription, and translation due to different distribution sites [15–19]. However, since G4s consist of G-rich sequences, the telomeric regions, gene promoters, and 5′UTR harboring these structures are highly susceptible to ROS-induced oxidative damage [20–22]. These findings suggest that G4s may be involved in ROS-mediated DNA damage. And further research found that H_2_O_2_-induced oxidative stress triggers the cytoplasmic accumulation of G4s, wherein they interact with some proteins to form “stress particles” that alter mRNA translation [23, 24]. Therefore, it is critical and meaningful to explore the relationship between ROS and G4s, which can improve our understanding of the regulation of DNA damage repair pathways, telomere shortening, antioxidant action, epigenetic modification, etc., in response to oxidative stress.

## Structural and functional characteristics of G4

The core of the G4 structure is the G-tetrad which consists of four G-rich chains connected by Hoogsteen hydrogen bonds. The different arrangements of the four chains lead to considerable variation in the structure and stability of G4s [11]. The structure, distribution, and regulatory role of G4 have been elucidated to a large extent through bioinformatics prediction, physical methods, sequencing technology, and molecular biology approach [25–27]. G4s are widely distributed in the nuclear, cytoplasmic, and mitochondrial chromatin, as confirmed using small molecule fluorescence probes and antibodies targeting BG4 and 1H6 [28–31]. Furthermore, G4s are enriched in the telomeres, promoter regions, exons, introns, and 3′UTR regions [32, 33], all of which are involved in the regulation of DNA replication, transcription, and protection of telomere termini [34]. G4-sequencing has further revealed that G4s are highly enriched in the promoters and TSSs of human and mouse genes [35]. Computational tools can help predict PQSs to gain further insights into the distribution of G4s in target genes [25, 36, 37]. Furthermore, the regulatory role of G4s can be validated in vivo and in vitro using G4 stabilizers [38–40]. However, G4s likely exist in a dynamic equilibrium due to external and internal factors interplay.

### Factors affecting G4 formation and stability

**Fig. 1:**
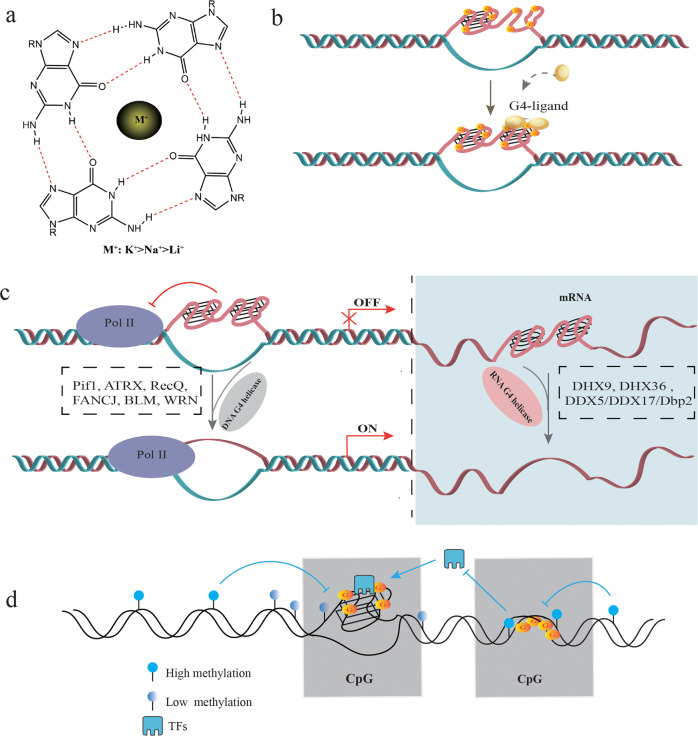
Factors affecting G4 structure formation and stability. **a** Due to the unique negative charge channels in the G4 structure, it is stabilized upon interacting with cations such as K^+^, Na^+^, and Li^+^ through electrostatic forces. **b** Small molecules and ligands enhance G4 structural stability. **c** Helicase is involved in regulating the transcription and translation of genes by unwinding G4s. DNA helicases accelerate the unwinding of promoter region G4s and promote the transcription of genes by RNA polymerase (Pol II). RNA G4s are unstable and can be easily unwound by RNA helicases, thereby facilitating the translation process. **d** Interaction of DNA methylation and G4s: although CpG methylation can block the formation of G4 structures in the region harboring CpG islands, it can recruit TFs to the G4 motifs formed in hypomethylated regions, thereby promoting gene replication and transcription. In addition, the G4s can also reverse CpG methylation and maintain the hypomethylated state.

Due to the unique negative charge channels, the G4 structure interacts with cations, including K^+^, Na^+^, and Li^+^ through electrostatic forces [41] (Fig. 1a). K^+^ has the strongest stabilizing effect on G4s, and high intracellular levels of K^+^ are conducive to forming G4s [42]. Thus, K^+^ is routinely used to induce G4 structure formation in vitro [43], which can be visualized by circular dichroism and gel mobility shift assay [44]. Low hydration and a high density of nucleotides are other prerequisites of G4 formation and stability [45]. In addition, small molecule ligands can also selectively bind to and further stabilize the G4 structure [46] (Fig. 1b). Examples include PDS, acridine (BRACO-19; AS1410; RHPS4), quinacridone (BOQ1; NCQ), porphyrin (TMPyP4; NMM), and alkaloids present in traditional Chinese medicine, such as berberine, isaindigotone, quinazoline, etc [47–50]. (Table 1). Especially, PDS, Braco-19, and TMPyP4 have been widely used to explore potential mechanism associated with G4 in aging-related diseases, metabolic diseases, and antiviral therapy [39, 44, 51–53].

**Table 1:** G4-ligand and its biological functions.

G4-ligand		Target gene	Region	Biological function	Cell model	Related diseases/application
Classification	Ligand					
	Pyridostatin [39, 51, 194]	ATG7, Brac1	Promoter Telomere	Antiviral, transcription inhibition DSB	Neural stem cell, progenitor cells, neurons and astrocytes, microglia, 293T cells	Zika virus, neurodegeneration, cancer
Acridine	BRACO-19 [39, 195, 196]	c-Myc, KRAS, hTERT	promoter Telomere	Telomere shortening, Antiviral, antiproliferative	Neurons, MRC-5, DU145 prostate cancer cells, uterus carcinoma cell	Viral infectious disease (HBV, HIV, HPV, HSV), neurodegeneration, cancer
	AS1410 [197]	Tel	Telomere	Telomere shortening, transcription inhibition, antiproliferative	MCF7, A549 lung cancer	Lung cancer, Breast Cancer
	RHPS4 [198–200]	Tel	Telomere	antiproliferative, DSB	Osteosarcoma Cells, brain tumor cells, astrocytoma cell	Osteosarcoma, astrocytoma
Quinacridine	BOQ1 [201]	c-Myc	Promoter	Transcription inhibition		
	MMQ [48, 202]	Tel	Telomere	Antiproliferative	A431 cell	
Qorphyrin	TMPyP4 [106, 195, 203–205]	BCL2, c-Myc, kRAS, PDGFa, VEGF, Tel	Promoter Telomere	Inhibit telomerase activity, anti-cancer	human endometrial cancer cell (HEC1A) Breast-Cancer Cells(MDA-MB231), human kidney cancer cell lines (A498 and 786O), Hela, Miapaca	HBV, cervical cancer, Breast Cancer, endometrial cancer, kidney cancer
	NMM [206–208]	P1192R, D117L	Promoter Telomere	Antivirals	293T cells, PAM Cells	African swine fever virus,G4-fluorescence probe
Traditional chinese medicine alkaloids	Berberine [50, 209–214]	DUX4, Tel, KRAS, c-Myc, relaxin-1, c-Kit	Promoter Telomere	Suppress gene expression, antiproliferation, inhibit telomerase activity, stimulates the unusual -1 frameshift, antifibrosis	Rhabdomyosarcoma (RD) TE671 cells, SW620 cells, Cardiac fibroblasts	facioscapulohumeral muscular dystrophy, G4-fluorescence probe, cardiac fibrosis
	Isaindigotone [215, 216]	c-Myc, Tel	Promoter Telomere	Antiproliferation, inhibit telomerase activity	SiHa cells, HL60 and CA46 cancer cells	G4-fluorescence probe
	Quinazoline/quinazoline derivatives [217, 218]	STAT3, Tel,c-Kit, hVEGF, c-Myc	Promoter Telomere 5′UTR	Antiproliferation, DSB, suppressed cell migration, induced apoptosis	HeLa Cells, HCC cells	
	Quercetin [219, 220]	c-Myc, Tel	Promoter Telomere	Apoptosis-mediated cell death,	HeLa cells	
Ellipticine analogs	N-TASQ [38, 221]				MCF7, B16F10 and U2OS cells	G4-fluorescence probe
	GQC-05 [222, 223]	Myc, BCL-X	Promoter	DSB and apoptosis, modulate splicing, apoptosis	AML cells, KG-1a cells, CMK cells and TF-1 cells	
	NSC311153 [224]	RET		Antiproliferation	papillary thyroid carcinoma cells	
Quinoxaline analogs	QN-1 [225]	c-Myc	Promoter	Cell cycle arrest and apoptosis, repressed metastasis, antiproliferation		
	DC-34 [226]	c-Myc	Promoter	Antiproliferation	Human multiple myeloma cell lines	
Fluoroquinolones	CX-3543 [227, 228]	CCAT1, c-Myc	Promoter	Replication defects, DSB, and telomere instability, apoptosis, antiproliferation	TNBC cell	Triple-negative breast cancer
	CX-5461 [194, 228–230]	c-Myc, Tel, c-Kit1	Promoter Telomere	Replication defects, DSB, and telomere instability,	breast cancer cell, HCT116 and U2OS cells, GBM cells.	Prostate cancer(phase I/II clinical trials), solid tumors with BRCA2 or PALB2 mutations(phase I clinical trials)

The unfolding and folding of G4s during replication or transcription are mainly catalyzed by helicases [54, 55] including DNA G4 helicases such as Pif1, ATRX, RecQ, FANCJ, BLM and WRN [54], and RNA G4 helicases such as DHX9, DHX36, and the DDX5/DDX17/Dbp2 subfamily [56] (Fig. 1c). Mutations in ATRX are associated with cognitive deficits, developmental abnormalities, and cancer. Treatment of ATRX-null neuro-progenitor cells with G4 ligand increased DNA damage [57]. DHX36 is an RNA helicase that binds to and unwinds pri-miR-26a RNA G4 structure and thus regulates miR-26a biogenesis and function in hepatic lipid metabolism and insulin sensitivity [44]. Overexpressing Pif1 in neurons rescued phenotypes associated with PDS treatment and increased autophagy [39]. Thus, targeted regulation of helicase function is a potential therapeutic strategy against cancer and aging-related neurological and metabolic diseases.

The stability of G4 structure is also affected by DNA methylation, a key epigenetic modification that acts as a transcriptional repressor [58]. DNA methylation mainly occurs on CpG dinucleotides enriched in G4-forming DNA sequences [14]. Computational analyses have shown that hypermethylated CpG tends to be associated with low G4 stability [59]. Furthermore, one study showed that the protein-binding ability of G4 DNA is significantly inhibited by CpG methylation [60]. The G4 structure forming in the human genome is strongly associated with CpG hypomethylation [61, 62] (Fig. 1d). These findings indicate that G4s may be closely related to epigenetic regulation, especially DNA methylation.

### Factors involved in G4-mediated biological functions

**Fig. 2:**
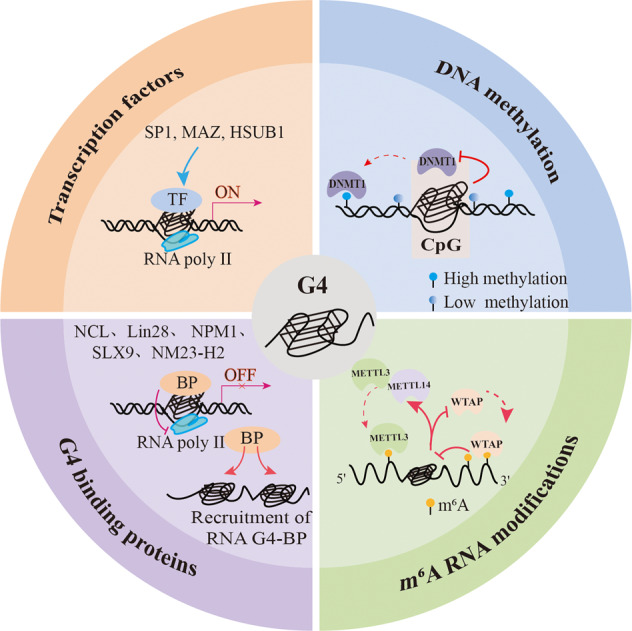
Factors involved in G4-regulated biological functions. The biological regulation functions of G4s can be affected by transcription factors (TFs), binding proteins(BP), DNA methylation modification, and m^6^A RNA modification.

The formation of G4 is strongly related to epigenetics and gene regulation. The regulatory role of G4s on gene transcription or translation are influenced by a variety of intracellular factors such as TFs, binding proteins, and epigenetic modifications (Fig. 2). Computational predictions indicated that TFs are strongly enriched in the G4 motifs of promoters across genomes of multiple species, and dozens of these TFs appear to be conserved [60, 63]. Genome-wide study showed that endogenous G4s are prominent binding sites of numerous TFs, particularly at the promoters of highly expressed genes [61], which is suggestive of regulatory interactions between TFs and G4 structures at gene promoters. For example, SP1, a zinc-finger TF, control the expression of many house-keeping genes by binding to CpG-rich sites [62]. One study using an SP1-chip showed that 87% of the SP1 binding sites overlapped with G4-forming sequences [64]. Interestingly, the SP1 binding site at the promoter of the oncogene c-Kit forms G4 structures and does not harbor the consensus binding sequence [65]. Similarly, other zinc-finger TFs, including MAZ and HSUB1, also promote gene transcription through G4s [66, 67].

G4-binding proteins also play a crucial role in transcriptional and translational regulation, thus regulating diverse biological processes. PARP-1 was the first G4-binding protein to be identified and mediates transcriptional regulation and telomere end protection in the human genome [68]. Similar to the TFs, G4-binding proteins affect gene transcription in a bidirectional manner. For example, nucleoside (NCL) repress c-Myc and LTR transcription by binding to G4 forming in the gene promoter region and stabilizing its structure [69, 70]. NM23-H2 is a regulatory protein that can bidirectionally regulate gene transcription and is directly involved in epigenetic modification in a G4-dependent manner. Luciferase reporter assay and chromatin immunoprecipitation showed that recombinant purified NM23-H2 interacted with the G4 motif in the c-Myc promoter with high affinity. Furthermore, overexpression of NM23-H2 in the human cancer cell lines HeLa S3 and A549 enhanced c-Myc promoter activity [71]. Another study showed that NM23-H2 is enriched in the hTERT promoter, which has a relatively high number of G4s [72]. It binds to the G4 within the hTERT promoter and recruits epigenetic modifiers such as REST, co-REST, and LSD1 to form an epigenetic repressor complex, thereby inhibiting hTERT gene transcription [73]. NM23-H2 silencing, on the other hand, leads to a marked increase in modified histones, including H3K4me2, H3K4me1, and H3K9ac [73].Therefore, the distribution characteristics of G4 in the promoter region and its binding relationship with TFs or binding proteins may determine its bidirectional regulation role in gene transcription. However, the specific regulation mechanism still needs to be further explored.

Methylation of the CpG islands in promoter regions is the main epigenetic mechanism of gene silencing [74]. The presence of methylated cytosines in the CpG-rich gene promoters blocks the binding of TFs, leading to gene silencing [75]. For example, CpG methylation of G4 oligonucleotides within promoter regions alters the binding of SP1 [76]. To some extent, the formation of G4 structures may influence the functions of TFs when hypomethylated. However, the specific mechanisms connecting G4-related TFs and epigenetic regulation need further exploration. Bioinformatics analyses have shown that G4s are also enriched in the CpG islands in genomic DNA[77, 78]. CpG nucleotides present within the PQS motifs are hypomethylated, and low methylation levels within promoters are closely associated with PQS [79]. There are reports that recombinant human DNMTs can bind to G4 DNA with high affinity in vitro, which could be the conduit through which DNMTs recognize DNA [80]. In addition, G4 binding inhibits the activity of DNMT1, and DNMT1 binding sites enriched in G4 structures are strongly hypomethylated in human leukemia cells, suggesting that DNMT1 may be sequestered at G4 sites to inhibit the methylation of proximal CpG island promoters [81].

The m^6^A modification regulates various aspects of RNA metabolism, including splicing, translation efficiency, nuclear export, stability, and translation [82]. Bioinformatics analyses have revealed significant overlap and functional synergy between G4 structures and m^6^A-modified sites [83, 84]. Although G4s can provide a framework for m^6^A modification, the latter can destabilize the G4 structure [85]. This suggests that m^6^A modifications likely regulate gene expression by controlling G4 formation. In fact, the presence of G4s can also affect m^6^A modification. For example, as a core component of the m^6^A methyltransferase complex, WTAP has an inhibitory effect at splice sites with potential G4 formation [86]. However, G4-forming regions in many DRACH sequences can directly recruit METTL3/METTL14 to specific methylation sites in their vicinity [87]. Taken together, the G4s at m^6^A-modified sites recruit m^6^A methyltransferase complex, and m^6^A destabilizes G4, which may affect RNA metabolism.

## ROS and G4S

As mentioned, G4s are highly susceptible to ROS-mediated oxidative damage due to their G-rich sequences. The roles of ROS in the formation, stabilization, and regulatory role of G4s will be discussed in the following sections.

### Role of ROS in G4 Formation and Stability

**Fig. 3:**
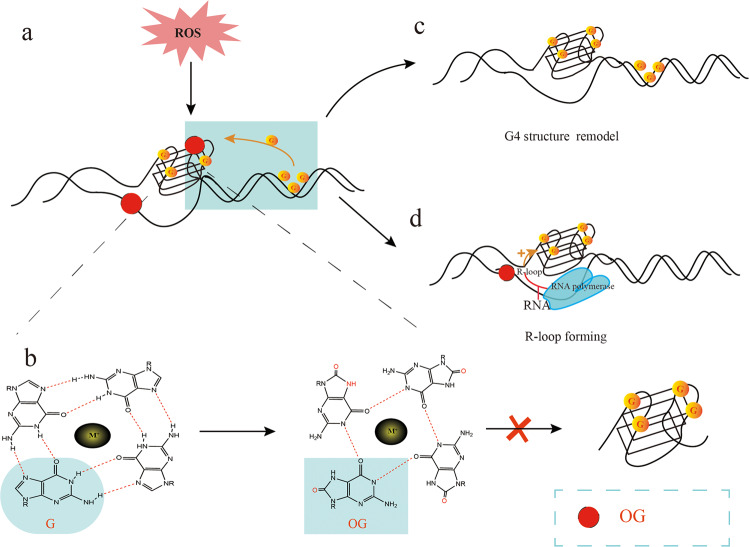
Role of ROS in G4 formation and stability. ROS oxidizes guanine in G4s and blocks the Hoogsteen hydrogen bonds, which disrupts the thermal stability of the G4 structure (**a**, **b**). The guanine bases in the surrounding G-rich region can compensate for the missing guanine and stabilize the G4 structure (**a**). In addition, G4 can also be modified into other structural forms by altering the G4-forming mode (**c**). Oxidation of the guanine base in the DNA template chain can also form OG. With the help of RNA polymerase, the RNA chain can form R-loop with the original DNA chain and stabilize the G4 structure (**d**).

ROS or free radicals such as O_2_^•−^, H_2_O_2,_ and ^•^OH are generated in both hypoxic and hyperoxic conditions [88]. H_2_O_2_ decomposes in the presence of Fe^2+^ into CO3^•−^ and HO^•^, of which the former is a highly potent oxidizer [89]. The G tracks of G4 structures are highly susceptible to one-electron oxidation by the CO3•−ions [90]. However, it is unclear whether ROS triggers the dissociation of G4s. ROS can reduce the thermal stability of G4 motifs by oxidizing guanine into OG, a common biomarker of oxidative stress [91, 92]. Oxidation of guanine prevents the formation of Hoogsteen hydrogen bonds, which obstructs G4 formation [93] (Fig. 3a, b). However, other studies have reported a stabilizing effect of OG on the G4s. For example, the OG in a G-quartet can be substituted for guanine in the outer G quartets of tetramolecular G4s, thereby increasing its stability [94] (Fig. 3a). Furthermore, recent models of the human telomere indicate a fifth G track, which could be exchanged with the OG-bearing track to maintain the stability of the fold [95]. Finally, the oxidation of some guanine bases can be compensated by surrounding guanine bases to maintain the stability of G4 [96]. Another study showed that OG could enhance the stability of the G4 structure located in the promoter region of the BCL2, which suggests a potential novel regulatory role of oxidative stress in general, and specifically in BCL2 gene transcription [97]. Thus, the native plasticity of G4s can accommodate structurally perturbing oxidative modifications (Fig. 3c).

On the other hand, ROS can also trigger the formation of G4s at transcriptionally active sites containing R-loops [98, 99] (Fig. 3d). Previous reports have demonstrated that ROS induces R-loop formation and facilitate recruitment of R-loop sensors such as RAD52 to the damaged sites [100]. In one study, oxidative damage triggered the formation of R-loop and G4 structures in a BLM helicase-dependent manner [98]. D-loops are located at the 3′ overhang of telomeres where G4 motifs are prevalent, and those harboring OG are the preferred substrate for Werner helicase (WRN) compared to the undamaged D-loops [101]. Thus, G4 structures may form more frequently in damaged R-loop or D-loops due to guanine oxidation.

ROS is generated in response to various environmental stimuli, including UV light, ionizing radiation, and chemicals such as hydrogen peroxide [102**]**. Recent studies show that G4s can directly produce guanine radicals induced by UV irradiation, leading to DNA damage [103, 104]. The mechanisms underlying G4-mediated ROS production in response to UV irradiation need further investigation. Interestingly, H_2_O_2_ can enhance TMPyP4-induced DNA damage and provoke stronger DDR in cancer cells but not in the normal cells [105]. TMPyP4 inhibits tumor proliferation by directly targeting G4 [106]. These results suggest that ROS induced by mild to moderate levels of H_2_O_2_ may accelerate DNA damage and transcriptional inhibition by facilitating the formation of G4s. Thus, targeting G4s in cancer cells may be more likely to inhibit the transcription of oncogenes with H_2_O_2_ stimuli.

To summarize, the equilibrium between the formation and distribution of G4s maintains normal cellular functions and also allows the G4s to adapt structurally in response to ROS.

### ROS regulates G4-mediated biological process

*ROS and telomeric G4s*. Telomeres, the terminal parts of chromosomes, are special structural regions that play an important role in the structure and stability of linear chromosomes [107]. Telomere length is a marker of cellular age, and aberrant telomere length is associated with carcinogenesis, aging, and age-related diseases [108–110]. The length of telomeres is controlled by genetic as well as environmental factors, including lifestyle factors, physiological stress, inflammation, oxidative stress, and carcinogens [111, 112], of which oxidative stress is the most potent endogenous driver of telomere shortening [111, 113].

**Fig. 4:**
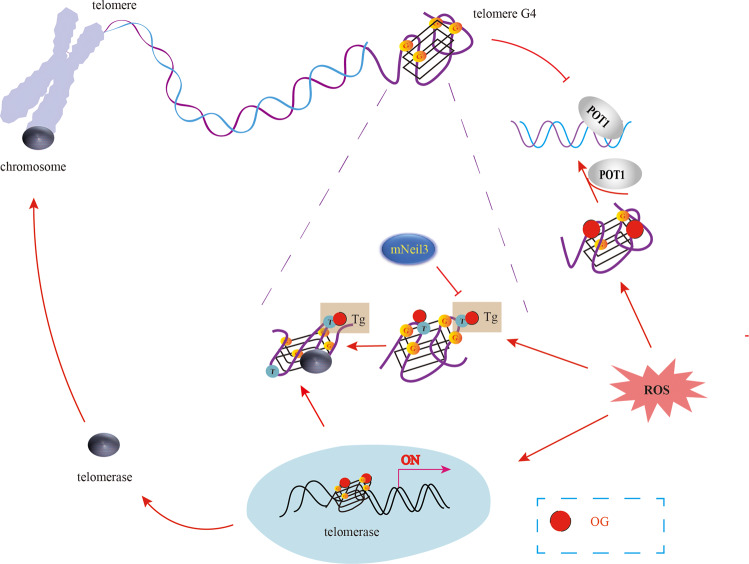
The role of G4 in ROS-mediated regulation of telomere function. OG can activate telomerase by destabilizing DNA G4 structures and inducing Tg structure at thymine (T), which alters the conformation of original G4 to enhance telomerase binding. Meanwhile, mNeil3 can inhibit the formation of Tg, thereby maintaining the stability of the original G4 structure. On the other hand, OG can reduce telomere G4 stability, unfold their structure, and reduce the rejection of POT1, thereby maintaining the integrity and function of DNA telomeres.

Telomeres are particularly susceptible to oxidative damage due to the presence of their G-rich sequence [114]. High levels of ROS also induce single-strand breaks (SSBs) at the telomeres, leading to telomere loss [115]. Secondly, the DDR is not activated at the telomeres, and the shelterin proteins TRF1 and TRF2 bind to ROS-damaged telomere DNA with low affinity [116, 117]. On the other hand, ROS can also promote telomere lengthening by increasing telomerase activity. For example, mild ROS elevation in tumor cells activates telomerase, accelerates telomere elongation, and promotes tumor cell proliferation [118]. However, the specific mechanisms through which ROS regulates telomeres length and function are not well understood. A new study found that G4 is beneficial to ROS-induced telomere lengthening [119] (Fig. 4). Oxidative DNA damage can increase telomerase activity by destabilizing DNA G4 structures [120]. Moreover, thymine glycol (Tg), one of the most common oxidative DNA damage bases, enhances telomerase activity and extension by disrupting the folding of telomeric G4s [120–122]. On the other hand, OG sites generated in the telomeric G4s under oxidative stress can significantly reduce their structural instability and induce unfolding, stabilizing the protection of telomeres 1 (POT1) at the G4s and maintaining telomeric integrity [120]. These findings also explain why uncleared OG causes telomere lengthening in OGG1-deficient mice and yeast [123].

Though ROS can repress telomeric G4 formation, human telomeric G4 is characterized by remarkable structural stability that confers resistance to oxidative stress. Telomeric G4 can produce one or even clustered OG lesions that can still form non-Hoogsteen hydrogen bonds with neighboring guanines [21]. This suggests that increasing G4 stability may increase the antioxidant capacity of telomeres and delay cellular senescence. However, although G-rich telomeric DNA is susceptible to oxidation, only a few specific repair enzyme for telomeric G4s have been identified so far. For example, Zhou et al. reported that while mNEIL3 can excise Tg from G4 DNA, none of the glycosylases (NEIL1, NEIL2, mNEIL3, NTH1, and OGG1) can repair OG residues in quadruplex DNA [124]. Hence, the role of G4 in regulating telomere function and repairing oxidative stress-induced telomere damage need further exploration.

#### ROS regulates transcription in a promoter G4-dependent manner

Low levels of ROS function as redox-signaling molecules in multiple pathways and promote protein expression by phosphorylating tyrosine residues, inhibiting phosphatases, and activating transcription factors. In fact, ROS-generating metabolic processes can directly oxidize guanine to OG, thereby affecting gene regulation [125]. A recent study demonstrated that guanine oxidation to OG is 3-fold higher in the PQS of gene promoters [126], and some of these regions have the propensity to form G4s [90]. For example, OG formation in the G4s of the PCNA promoter increased gene expression [127, 128]. The G4s of NTHL1 promoters show similar functions [126]. In addition, oxidative damage in a promoter G4 can also upregulate gene expression by guiding the DNA repair process to the regulatory region depending on the position of the PQS in the promoter [126]. Established G4s occur naturally in a similar location relative to the TSS for possible oxidation-induced gene activation [129].

**Fig. 5:**
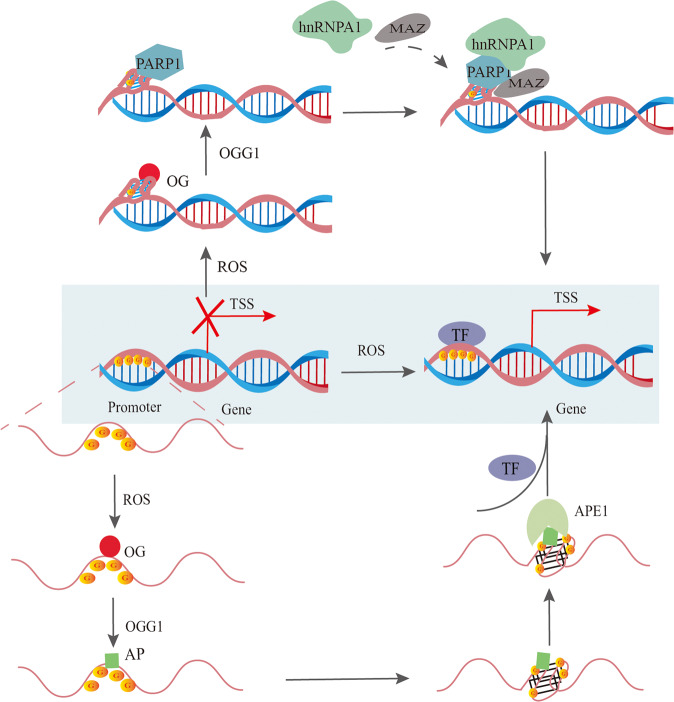
The possible mechanisms of ROS-regulated gene transcription depending on promoter G4s. Under oxidative stress, ROS can lead to OG formation in the G-rich region of promoters, which recruits OGG1 to clear OG and form the AP site. APE1 then binds to AP when looped out in the G4 fold to recruit TFs. On the other hand, ROS can also directly attack the G4 structure of the promoter region and form OG, which facilitates the recruitment of PARP1 and the binding of other transcriptional activating proteins to promote gene expression.

The mechanisms through which ROS regulates gene transcription via G4 motifs of promoters are summarized (Fig. 5). The ROS-induced OG in the PQS is removed by OGG1, which yields a duplex-destabilizing AP that allows a structural switch to the G4 fold. The apurinic/apyrimidinic endonuclease 1 (APE1) binds to AP when it is looped out in the G4 fold to activate transcription factors such as HIF-1α and AP-1, resulting in gene transcription when the modifications are close to the TSS [127, 130]. This hypothesis was made based on a previous study on the mechanics of VEGF activation [126]. Activation of NEIL3 expression through this proposed mechanism allows cells to respond to mutagenic DNA damage induced by oxidative or inflammatory stress [131].

In addition, Chip-qPCR experiments have shown that OG is more abundant in G4 than in the non-G4 regions in the KRAS promoter. OGG1 is recruited to the KRAS promoter following H_2_O_2_-induced guanine oxidation, removing OG from the G4s. Especially, Furthermore, the OG favors recruitment of the G4s to the promoter of MAZ and hnRNP A1 [132]. The same team also found that H_2_O_2_ recruits poly [ADP-ribose] polymerase 1 (PARP-1) to KRAS promoter region, wherein it binds to local G4 structures, undergoes auto PARylation and activates KRAS transcription by recruiting MAZ and hnRNPA1 [132, 133] (Fig. 5). In fact, the G4 forming sequence in the promoter of the proto-oncogene c-kit stimulates the enzymatic activity of PARP-1, which is dependent on the loop features and oxidative damage [134]. However, it remains to be verified whether PARP-1 binding to the c-Kit promoter G4s recruits other binding proteins to activate gene transcription. Taken together, although G4 has been shown to regulate gene transcription bidirectionally in previous studies, it can also indirectly activate transcription by promoting base repair and recruiting transcriptional activator proteins in a ROS-dependent manner.

## G4s regulate the antioxidant system

### G4 and stress granules

Stress granules (SGs) are dense granules formed in the cytoplasm of eukaryotic cells in response to oxidative stress, heat shock, hypoglycemia, and hypoxia [24]. It consists of mRNA in the translation initiation stage, RNA-binding proteins, and non-RNA-binding proteins, although the exact composition depends on the stress mode [24]. In fact, its constituent structure involves multiple TFs and other binding proteins such as eIF protein family (eIF2, eIF4G), T-cell intracellular antigen1 (TIA1), Y-box binding protein 1 (YB-1), USP10 and G3BP1 [135–138]. Therefore, SGs are crucial to the antioxidant response, enhance mRNA stability and translation.

**Fig. 6:**
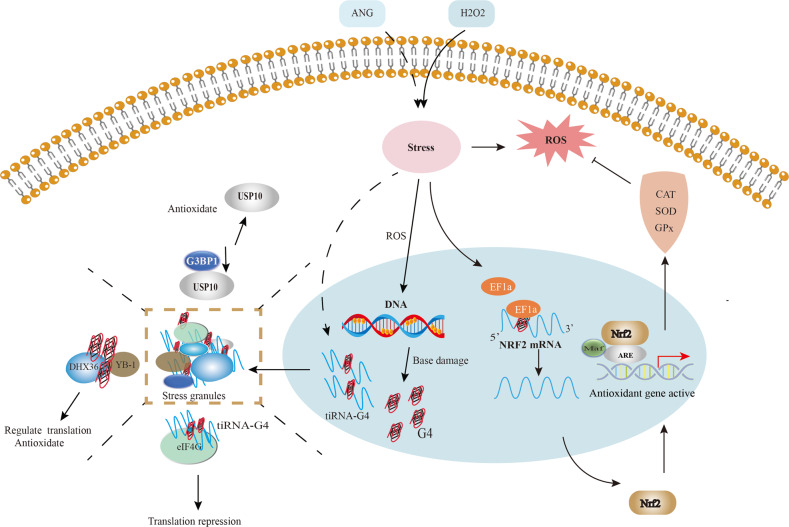
Crosstalk between Nrf2, stress particles, and G4. H_2_O_2_-induced ROS promotes Nrf2 protein translation via EF1a interaction with the G4 in Nrf2 5′UTR. Activating downstream antioxidant genes enhances antioxidant capacity and protects normal cells from oxidative damage. Stress-induced DNA damage promotes the formation of DNA-G4 and tiRNA-G4 complex, which trigger SGs assembly by recruiting cytosolic proteins, which involved in antioxidant response and mRNA translation.

Increasing evidences have shown that G4s play important roles in the formation and functional regulation of SGs following different stress stimuli (Fig. 6). For example, Cells treated with a mild dose of H_2_O_2_ show a significant increase in cytosolic G4s, which co-localize SH-related marker proteins [23]. H_2_O_2_-induced ROS not only causes DNA damage, but also produces single-stranded DNA that is more likely to form G4s. The latter then binds to YB-1, the major component of SGs, and promotes SGs assembly in the presence of DHX36 [23, 139]. YB-1 is a translation-regulating protein that can promote the formation of SGs [140]. Knocking down YB-1 expression in tumor cells inhibited G3BP1 translation, which prevented the formation of SGs and sensitized the cells to oxidative stress, thereby inhibiting their proliferation and invasion [140, 141]. Thus, endogenous G4s link oxidative stress-induced DNA damage to translation via the formation of SGs. In addition to stabilizing SGs, G4s can also transiently inhibit translation under stress. One study showed that tRNA-derived RNA fragments (tiRNA) are formed along with G4s during stress-induced angiogenin(ANG), promoting SGs assembly and inhibiting translation [142]. In addition, eIF4G, a protein that plays an important role in the initiation of eukaryotic translation, directly binds to the G4s and represses tiRNA-mediated translation. The HEAT1 region of the eIF4G protein is the main binding region of the G4-tiRNA complex [143]. These findings provide a new perspective for us to further explore the regulatory mechanism of translation under stress.

The formation of SGs are associated with neurological diseases, myopathies, and tumors [144–146]. Thus, developing targeted drugs stabilizing SGs have been a new promising direction in cancer therapeutics [147**]**. With an increased understanding of the regulatory role of G4s in the process of SGs forming, drugs targeting G4s also have a new potential clinical value.

### G4 and Nrf2

Low to moderate levels of oxidative stress activate antioxidant and detoxification genes that contain the antioxidant response element (ARE) in their promoters, which is the binding site of the Nrf2 transcription factor [148, 149]. The Nrf2/ARE signaling pathway is the major cellular defense against exogenous oxidative damage. Without any stimulus, Nrf2 binds to the cytoplasmic chaperone kelch-like Ech-associated protein 1 (Keap1) and is sequestered in the cytoplasm relatively inhibited. When exposed to oxidative stress, Nrf2 uncouples from Keap1. It is translocated to the nucleus, wherein it binds to the AREs along with the Maf protein, and initiates the transcription of phase II detoxification enzymes and antioxidant genes, such as heme oxygenase-1 (HO-1), CAT, SOD, GSTs, NADP (H) quinone oxidoreductase 1 (NQO1), etc [150, 151].

A recent study revealed a potential parallel intramolecular G4 forming sequence in the promoter region of Nrf2 in the presence of K^+^, which is close to several putative TF-binding sites. This suggests the presence of a natural G4 structure in the promoter region of Nrf2 [152], which raises the possibility for targeted inhibition of Nrf2 transcription through G4 ligands. In addition to DNA G4s, RNA G4s are also involved in several biological processes, such as telomere homeostasis, mRNA localization, 3-terminal processing, alternative splicing, and translation regulation [153]. For instance, the G4 structure in the 5′UTR plays a regulatory role in translation [154]. The 5′UTR of Nrf2 mRNA can form G4, which interacts with EF1a and promotes de novo Nrf2 protein translation under H_2_O_2_ stress [155]. These results suggest that the G4s of Nrf2 mRNA may assist Nrf2 protein translation under oxidative stress by recruiting related binding proteins, which thus activate the cellular antioxidant response (Fig. 6). Multiple genes such as P62, PI3K, KRAS, B-Raf, and c-Myc regulate Nrf2 expression in different cells [156–159]. Although G4 structures have been confirmed in these genes’ promoter regions or mRNAs, it is unclear whether they also affect the transcription or translation of Nrf2 [40, 160, 161]. An in-depth exploration of the distribution and function of G4s in the oxidation and antioxidant genes and the development of small molecules targeting G4s will allow the precise regulation of the antioxidant system in cancer and other pathological states.

## Potential involvement Of G4 In Ros-related diseases

### Cancer

**Fig. 7:**
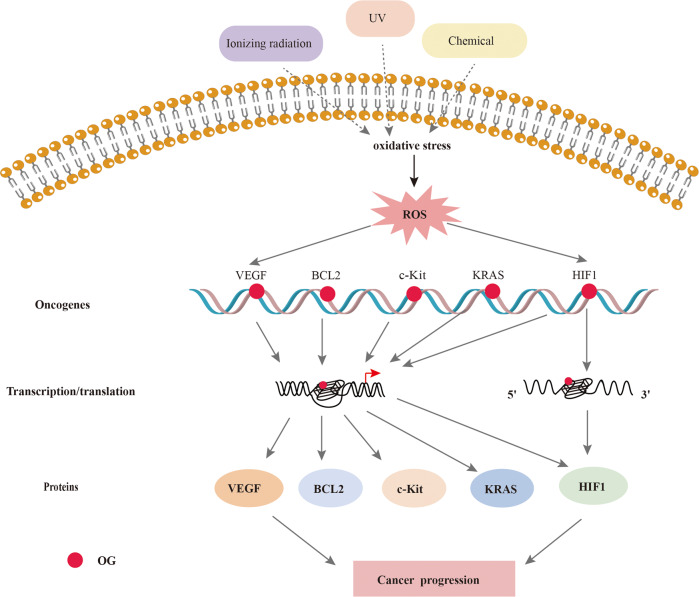
ROS regulates oncogene expression depend on G4. The ROS in cancer cells oxidizes guanine residues in the oncogene promoter region or mRNA G4s to OG, which activates the G4-mediated DDR pathway and translation. The transcription and translation of oncogenes promot cancer development.

ROS activate oncogenic signaling pathways, enhance cancer cell survival and proliferation, drive DNA damage and genetic instability, and induce chemoresistance [162–164]. Recent studies have shown that G4s upstream of the promoter regions of proto-oncogenes can regulate their transcription [32, 164], and c-Myc, c-Kit, KRAS, Bcl-2, VEGF, and PDGF are some of the proto-oncogenes harboring G4 structures in their promoters [17, 132, 165–167]. Furthermore, under oxidative stress, G4s transcriptionally activate several oncogenes, such as VEGF, KRAS, and HIF1α. VEGF is overexpressed in various human cancers and is associated with poor prognosis. In an in vitro study, ROS increased the transcriptional activity of VEGF via its promoter G4, which was blocked in the presence of a G4 ligand [168]. HIF1α is a cancer-associated TF frequently activated in multiple cancers and has been implicated in ROS-induced carcinogenesis [169]. G4 structures are present in the HIF1α promoter and the 5′UTR [170, 171] and inhibit HIF1α transcription by blocking AP2 binding [172]. ROS also promotes the transcription of proto-oncogenes, such as BCL2, KRAS, and c-Kit, via G4s (Fig. 7). Although G4s seem indispensable to ROS-induced oncogene expression, the functions of oxidized G4s in stress response regulation in cancers are not entirely clear.

Photodynamic therapy (PDT) is a minimally invasive cancer treatment compared to surgery, chemotherapy, and radiotherapy [173]. Double-stranded DNA (dsDNA) is one of the potential targets of PDT [174]. During PDT, photosensitizer molecules accumulate in diseased tissues. They are activated by light or laser irradiation at a specific wavelength, producing ROS that triggers tumor cell death via apoptosis or necrosis [175]. Therefore, the characteristics of photosensitizers significantly affect the outcomes of PDT. A recent study showed that poly-G4-TMPyP4 complexes generate high levels of singlet oxygen at cancer lesions in response to UV irradiation and promote apoptosis of tumor cells [176]. TMPyP4 also induced photo-induced toxicity in HeLa cells and was shown to bind to the 3′-end of the KRAS G4s [177]. The promoters and 5′UTRs of HRAS and NRAS may also bind to TMPyP4 [178]. Other study showed that TMPipEOPP selectively binds to telomeric DNA G4 and helps to cleave the DNA chain upon photo-irradiation through ROS production, resulting in cancer cell death [179]. Since TMPipEOPP has little to no cytotoxicity in the absence of light, it could be an efficient photosensitizer for PDT. ZnP1 is a cationic porphyrin derivative that binds to the DNA groove [180**]**. It also binds selectively to the telomeric DNA G4 and generates singlet oxygen post-irradiation, which then oxidizes and cleaves guanine residues in the G4 and region. ZnAPC and TMPyP4-C14 have shown similar effects [181, 182] (Table 2). Thus, G4 ligands are promising photosensitizers for targeted PDT.

**Table 2:** G4-targeting photosensitizers.

	G4 ligand	Target G4	Gene	Cell type
Porphyrin derivatives	TMPyP4 [105, 177, 182, 231]	DNA	KRAS	MCF-7 human breast cancer cells、HeLa cells
	TMPipEOPP [179]	DNA		human colon carcinoma cells (HCT-8)
	ZnP1 [180]	DNA		
	TMPyP4-C14 [106]	RNA	KRAS, NRAS	pancreatic cancer cells
Phthalocyanine derivatives	ZnAPC [181]	RNA	NRAS	MCF-7 human breast cancer cells

### Neurodegenerative diseases

**Fig. 8:**
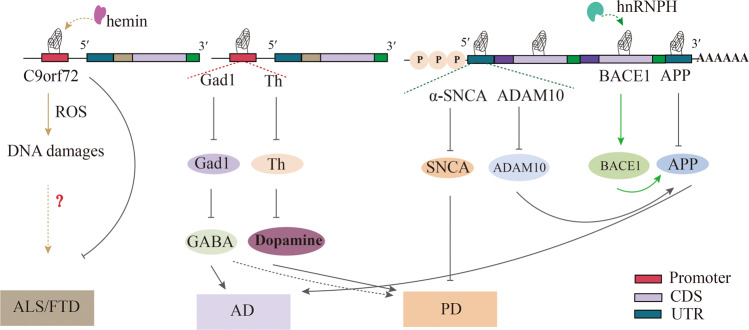
Proposed involvement of G4 in Neurodegenerative diseases. As a key pathogenic gene of ALS/FTD, G4-forming in the promoter region of C9orf72 can inhibit its transcription. In particular, G4s also recruits hemin, leading to DNA oxidative damage. Formation of G4s in the promoter of Gad1 and Th inhibit their transcription and further affects the synthesis of GABA and Dopamine. G4 formed in the 5′UTR region of α -SnCA, ADAM mRNA, and the 3′UTR region of APP mRNA can directly inhibit translation. However, G4 formed in the BACE1 mRNA 3′UTR region can recruit heterogeneous nuclear ribonucleoprotein H (hnRNPH), promote its translation, and induce the formation of APP, which are involved in the pathogenesis of AD and PD.

G4s are involved in various neurodegenerative diseases such as Alzheimer’s disease (AD) and Parkinson’s disease (PD) (Fig. 8). The G4s located in the promoter region of neuronal genes like C9orf72, glutamic decarboxylase 1 (Gad1) and tyrosine hydroxylase (Th) play a key regulatory role in their transcription [183–185]. In addition, the presence of G4s in the 3′UTR and 5′UTR regions of the mRNAs of Aβ precursor protein (APP), ADAM10, and α-synuclein (SNCA) is negatively correlated with the progression of AD and PD [186–188].

Oxidative stress is a regulatory element in aging and various neurological disorders [189], associated with excessive ROS production [190]. Interestingly, brain tissue samples from aged mice are enriched in G4-DNA structures absent in the brain tissues of young mice [39]. The guanines are frequently oxidized in aged cells and stabilize the G4 structure, making these non-canonical structures an attractive therapeutic target for neurodegenerative disorders [94].

The presence of G4s in the G-rich regions of the C9orf72 gene may recruit cellular hemin to form G4/hemin DNAzyme, which is associated with oxidative damage during neuronal degeneration [191] (Fig. 8). In the presence of hemin, telomeres can fold into G4 structures with catalytic oxidation properties in vivo [192]. Although there is no direct evidence that telomeric G4s enhance the peroxidase activity of hemin, the G4 ligand PhenDC3 displaces G4-bound heme in vitro. It induces HO-1 transcription, which indirectly supports the hypothesis that G4s sequester heme in the cell [193].

## Conclusions and perspectives

G4s are involved in regulating telomere length, gene transcription, translation, and epigenetic changes in the chromatin. The formation, stability and regulatory role of G4 structure are affected by various intracellular factors and epigenetic modifications. The unique structure and distribution of G4s provide new insights into the role of oxidative stress in regulating gene expression. ROS can affect the formation or dissociation of G4 motifs by reducing their thermal stability, altering the original G4 structural pattern, and influencing other chromatin structures at transcriptionally active sites such as R-loops and D-loops. G4s located at transcriptionally active sites are involved in base repair after ROS-induced DNA damage and thus promote gene transcription by recruiting associated proteins and TFs. Furthermore, G4s located in the 5′UTR or 3′UTR regions of mRNA are also involved in regulating translation under oxidative stress. These findings suggest that G4s are critical to ROS-mediated gene transcription and translation. In addition, G4s can improve ROS clearance and protect DNA from unwanted ROS by promoting the formation of SGs in the cytoplasm or activating Nrf2 translation. Thus, G4s may also be involved in cancer, aging, and degenerative disorders that are closely related to excessive ROS production and are promising therapeutic targets for these disorders.

Nevertheless, the current research on G4s are mainly descriptive, and the mechanisms underlying the regulation of G4s in response to ROS are largely unknown and need to be elucidated. For example, the optimal levels of intracellular ROS that regulate G4 formation and stability need further exploration. Following physical or chemical stimulation, whether ROS enhances G4-mediated tumor killing is not yet known. In additional, as mentioned above, G4s are involved in the formation of SGs, However, the specific role of G4 in regulating the biological function of SGs needs further experimental verification. In sum, the further exploration of these questions will contribute to understand the relationship between ROS and G4, and then provide some ideas for finding the new mechanism of G4-mediated biological regulation.

## Data Availability

All data generated or analyzed during this study are included in this published article.

## References

[1] Bouchez C, Devin A (2019). Mitochondrial biogenesis and mitochondrial reactive oxygen species (ROS): a complex relationship regulated by the cAMP/PKA signaling pathway. Cells.

[2] Sies H, Jones DP (2020). Reactive oxygen species (ROS) as pleiotropic physiological signalling agents. Nat Rev Mol Cell Biol.

[3] Zhao N, Ding B, Zhang Y, Klockow JL, Lau K, Chin FT (2020). Reactive oxygen species and enzyme dual-responsive biocompatible drug delivery system for targeted tumor therapy. J Control Release.

[4] Milkovic L, Cipak Gasparovic A, Cindric M, Mouthuy P-A, Zarkovic N (2019). Short overview of ROS as cell function regulators and their implications in therapy concepts. Cells.

[5] Huang X, Sheu G, Chang K, Huang Y, Hung P, Tsai N (2020). Pogostemon cablin triggered ROS-induced DNA damage to arrest cell cycle progression and induce apoptosis on human hepatocellular carcinoma in vitro and in vivo. Molecules (Basel, Switz).

[6] Neeley WL, Essigmann JM (2006). Mechanisms of formation, genotoxicity, and mutation of guanine oxidation products. Chem Res Toxicol.

[7] Krokan HE, Bjoras M (2013). Base excision repair. Cold Spring Harb Perspect Biol.

[8] Jackson SP, Bartek J (2009). The DNA-damage response in human biology and disease. Nature.

[9] Dinçer Y, Akkaya Ç, Mutlu T, Yavuzer S, Erkol G, Bozluolcay M (2019). DNA repair gene OGG1 polymorphism and its relation with oxidative DNA damage in patients with Alzheimer’s disease. Neurosci Lett.

[10] Baek J, Lee MG (2016). Oxidative stress and antioxidant strategies in dermatology. Redox Rep.

[11] Huppert JL (2008). Four-stranded nucleic acids: structure, function and targeting of G-quadruplexes. Chem Soc Rev.

[12] Falabella M, Fernandez RJ, Johnson FB, Kaufman BA (2019). Potential roles for G-quadruplexes in mitochondria. Curr Med Chem.

[13] Fleming AM, Zhu J, Ding Y, Visser JA, Zhu J, Burrows CJ (2018). Human DNA repair genes possess potential G-quadruplex sequences in their promoters and 5’-untranslated regions. Biochemistry.

[14] Huppert JL, Balasubramanian S (2007). G-quadruplexes in promoters throughout the human genome. Nucleic Acids Res.

[15] Neidle S (2010). Human telomeric G-quadruplex: the current status of telomeric G-quadruplexes as therapeutic targets in human cancer. FEBS J.

[16] Kim N (2019). The interplay between G-quadruplex and transcription. Curr Med Chem.

[17] Salvati E, Zizza P, Rizzo A, Iachettini S, Cingolani C, D’Angelo C (2014). Evidence for G-quadruplex in the promoter of vegfr-2 and its targeting to inhibit tumor angiogenesis. Nucleic Acids Res.

[18] Varshney D, Spiegel J, Zyner K, Tannahill D, Balasubramanian S (2020). The regulation and functions of DNA and RNA G-quadruplexes. Nat Rev Mol Cell Biol.

[19] Reina C, Cavalieri V. Epigenetic modulation of chromatin states and gene expression by G-quadruplex structures. Int J Mol Sci. 2020;21:4172. 10.3390/ijms21114172 PMC7312119 32545267

[20] Clark DW, Phang T, Edwards MG, Geraci MW, Gillespie MN (2012). Promoter G-quadruplex sequences are targets for base oxidation and strand cleavage during hypoxia-induced transcription. Free Radic Biol Med.

[21] Miclot T, Corbier C, Terenzi A, Hognon C, Grandemange S, Barone G (2020). Forever young: structural stability of telomeric guaninequadruplexes in presence of oxidative DNA lesions. Chemistry.

[22] Szalai VA, Singer MJ, Thorp HH (2002). Site-specific probing of oxidative reactivity and telomerase function using 7,8-dihydro-8-oxoguanine in telomeric DNA. J Am Chem Soc.

[23] Byrd AK, Zybailov BL, Maddukuri L, Gao J, Marecki JC, Jaiswal M (2016). Evidence that G-quadruplex DNA accumulates in the cytoplasm and participates in stress granule assembly in response to oxidative stress. J Biol Chem.

[24] Protter DSW, Parker R (2016). Principles and properties of stress granules. Trends Cell Biol.

[25] Puig Lombardi E, Londoño-Vallejo A (2020). A guide to computational methods for G-quadruplex prediction. Nucleic Acids Res.

[26] Chambers VS, Marsico G, Boutell JM, Di Antonio M, Smith GP, Balasubramanian S (2015). High-throughput sequencing of DNA G-quadruplex structures in the human genome. Nat Biotechnol.

[27] Biffi G, Tannahill D, McCafferty J, Balasubramanian S (2013). Quantitative visualization of DNA G-quadruplex structures in human cells. Nat Chem.

[28] Zhang S, Sun H, Chen H, Li Q, Guan A, Wang L (2018). Direct visualization of nucleolar G-quadruplexes in live cells by using a fluorescent light-up probe. Biochim Biophys Acta Gen Subj.

[29] Xu S, Li Q, Xiang J, Yang Q, Sun H, Guan A (2015). Directly lighting up RNA G-quadruplexes from test tubes to living human cells. Nucleic Acids Res.

[30] Liu S, Bu L, Zhang Y, Yan J, Li L, Li G (2021). Subtle structural changes of dyes lead to distinctly different fluorescent behaviors in cellular context: the role of G-quadruplex DNA interaction using coumarin-quinazolinone conjugates as a case study. Anal Chem.

[31] Kazemier HG, Paeschke K, Lansdorp PM (2017). Guanine quadruplex monoclonal antibody 1H6 cross-reacts with restrained thymidine-rich single stranded DNA. Nucleic Acids Res.

[32] Hansel-Hertsch R, Beraldi D, Lensing SV, Marsico G, Zyner K, Parry A (2016). G-quadruplex structures mark human regulatory chromatin. Nat Genet.

[33] Blasco MA (2005). Telomeres and human disease: ageing, cancer and beyond. Nat Rev Genet.

[34] Rhodes D, Lipps HJ (2015). G-quadruplexes and their regulatory roles in biology. Nucleic Acids Res.

[35] Marsico G, Chambers VS, Sahakyan AB, McCauley P, Boutell JM, Antonio MD (2019). Whole genome experimental maps of DNA G-quadruplexes in multiple species. Nucleic Acids Res.

[36] Brazda V, Kolomaznik J, Lysek J, Bartas M, Fojta M, Stastny J (2019). G4Hunter web application: a web server for G-quadruplex prediction. Bioinformatics.

[37] Kikin O, D’Antonio L, Bagga PS (2006). QGRS Mapper: a web-based server for predicting G-quadruplexes in nucleotide sequences. Nucleic Acids Res.

[38] Lejault P, Moruno-Manchon JF, Vemu SM, Honarpisheh P, Zhu L, Kim N (2020). Regulation of autophagy by DNA G-quadruplexes. Autophagy.

[39] Moruno-Manchon JF, Lejault P, Wang Y, McCauley B, Honarpisheh P, Morales Scheihing DA (2020). Small-molecule G-quadruplex stabilizers reveal a novel pathway of autophagy regulation in neurons. Elife.

[40] Wang W, Hu S, Gu Y, Yan Y, Stovall DB, Li D (2020). Human MYC G-quadruplex: from discovery to a cancer therapeutic target. Biochim Biophys Acta Rev Cancer.

[41] Sen D, Gilbert W (1990). A sodium-potassium switch in the formation of four-stranded G4-DNA. Nature.

[42] Palmer BF, Clegg DJ (2016). Physiology and pathophysiology of potassium homeostasis. Adv Physiol Educ.

[43] Vorlíčková M, Kejnovská I, Sagi J, Renčiuk D, Bednářová K, Motlová J (2012). Circular dichroism and guanine quadruplexes. Methods.

[44] Liu G, Du W, Xu H, Sun Q, Tang D, Zou S (2020). RNA G-quadruplex regulates microRNA-26a biogenesis and function. J Hepatol.

[45] Miller MC, Buscaglia R, Chaires JB, Lane AN, Trent JO (2010). Hydration is a major determinant of the G-quadruplex stability and conformation of the human telomere 3’ sequence of d(AG3(TTAG3)3). J Am Chem Soc.

[46] Curr Pharm DesVy Thi LeT, Han S, Chae J, HJCpd Park (2012). G-quadruplex binding ligands: from naturally occurring to rationally designed molecules. Curr Pharm Des.

[47] Xiong YX, Huang ZS, Tan JH (2015). Targeting G-quadruplex nucleic acids with heterocyclic alkaloids and their derivatives. Eur J Med Chem.

[48] Hounsou C, Guittat L, Monchaud D, Jourdan M, Saettel N, Mergny JL (2007). G-quadruplex recognition by quinacridines: a SAR, NMR, and biological study. ChemMedChem.

[49] Ramos CIV, Monteiro AR, Moura NMM, Faustino MAF, Trindade T, Neves M (2021). The interactions of H2TMPyP, analogues and its metal complexes with DNA G-quadruplexes—an overview. Biomolecules..

[50] Ciszewski L, Lu-Nguyen N, Slater A, Brennan A, Williams HEL, Dickson G (2020). G-quadruplex ligands mediate downregulation of DUX4 expression. Nucleic Acids Res.

[51] Zou M, Li J, Zhang M, Li J, Huang J, You P (2021). G-quadruplex binder pyridostatin as an effective multi-target ZIKV inhibitor. Int J Biol Macromol.

[52] Rodriguez R, Miller KM, Forment JV, Bradshaw CR, Nikan M, Britton S (2012). Small-molecule–induced DNA damage identifies alternative DNA structures in human genes. Nat Chem Biol.

[53] Chen J, Jin X, Mei Y, Shen Z, Zhu J, Shi H (2021). The different biological effects of TMPyP4 and cisplatin in the inflammatory microenvironment of osteosarcoma are attributed to G-quadruplex. Cell Prolif.

[54] Mendoza O, Bourdoncle A, Boule JB, Brosh RM, Mergny JL (2016). G-quadruplexes and helicases. Nucleic Acids Res.

[55] Sauer M, Paeschke K (2017). G-quadruplex unwinding helicases and their function in vivo. Biochem. Soc Trans.

[56] Caterino M, Paeschke K (2022). Action and function of helicases on RNA G-quadruplexes. Methods..

[57] Watson LA, Solomon LA, Li JR, Jiang Y, Edwards M, Shin-ya K (2013). Atrx deficiency induces telomere dysfunction, endocrine defects, and reduced life span. J Clin Investig.

[58] Jones PA (2012). Functions of DNA methylation: islands, start sites, gene bodies and beyond. Nat Rev Genet.

[59] Jara-Espejo M, Line SR (2020). DNA G-quadruplex stability, position and chromatin accessibility are associated with CpG island methylation. FEBS J.

[60] Bidula S (2021). Analysis of putative G-quadruplex forming sequences in inflammatory mediators and their potential as targets for treating inflammatory disorders. Cytokine.

[61] Spiegel J, Cuesta SM, Adhikari S, Hansel-Hertsch R, Tannahill D, Balasubramanian S (2021). G-quadruplexes are transcription factor binding hubs in human chromatin. Genome Biol.

[62] Narayan VA, Kriwacki RW, Caradonna JP (1997). Structures of zinc finger domains from transcription factor Sp1. Insights into sequence-specific protein-DNA recognition. J Biol Chem.

[63] Kumar P, Yadav VK, Baral A, Kumar P, Saha D, Chowdhury S (2011). Zinc-finger transcription factors are associated with guanine quadruplex motifs in human, chimpanzee, mouse and rat promoters genome-wide. Nucleic Acids Res.

[64] Raiber EA, Kranaster R, Lam E, Nikan M, Balasubramanian S (2012). A non-canonical DNA structure is a binding motif for the transcription factor SP1 in vitro. Nucleic Acids Res.

[65] Da Ros S, Nicoletto G, Rigo R, Ceschi S, Zorzan E, Dacasto M (2020). G-quadruplex modulation of SP1 functional binding sites at the KIT proximal promoter. Int J Mol Sci.

[66] Cogoi S, Paramasivam M, Membrino A, Yokoyama KK, Xodo LE (2010). The KRAS promoter responds to Myc-associated zinc finger and poly(ADP-ribose) polymerase 1 proteins, which recognize a critical quadruplex-forming GA-element. J Biol Chem.

[67] Gao J, Zybailov BL, Byrd AK, Griffin WC, Chib S, Mackintosh SG (2015). Yeast transcription co-activator Sub1 and its human homolog PC4 preferentially bind to G-quadruplex DNA. Chem Commun (Camb).

[68] Soldatenkov VA, Vetcher AA, Duka T, Ladame S (2008). First evidence of a functional interaction between DNA quadruplexes and Poly(ADPribose) polymerase-1. ACS Chem Biol.

[69] González V, Guo K, Hurley L, Sun D (2009). Identification and characterization of nucleolin as a c-myc G-quadruplex-binding protein. J Biol Chem.

[70] Tosoni E, Frasson I, Scalabrin M, Perrone R, Butovskaya E, Nadai M (2015). Nucleolin stabilizes G-quadruplex structures folded by the LTR promoter and silences HIV-1 viral transcription. Nucleic Acids Res.

[71] Thakur RK, Kumar P, Halder K, Verma A, Kar A, Parent JL (2009). Metastases suppressor NM23-H2 interaction with G-quadruplex DNA within c-MYC promoter nuclease hypersensitive element induces c-MYC expression. Nucleic Acids Res.

[72] Yadav V, Thakur R, Eckloff B, Baral A, Singh A, Halder R, et al. Promoter-proximal transcription factor binding is transcriptionally active when coupled with nucleosome repositioning in immediate vicinity. 2014;42:9602–11. 10.1093/nar/gku596 PMC4150765 25081206

[73] Saha D, Singh A, Hussain T, Srivastava V, Sengupta S, Kar A (2017). Epigenetic suppression of human telomerase (hTERT) is mediated by the metastasis suppressor NME2 in a G-quadruplex-dependent fashion. J Biol Chem.

[74] Ehrlich M (2002). DNA methylation in cancer: too much, but also too little. Oncogene.

[75] Heberle E, Bardet AF (2019). Sensitivity of transcription factors to DNA methylation. Essays Biochem.

[76] Yoshida W, Saito T, Yokoyama T, Ferri S, Ikebukuro K (2013). Aptamer selection based on G4-forming promoter region. PLoS ONE.

[77] Yoshida W, Saikyo H, Nakabayashi K, Yoshioka H, Bay DH, Iida K (2018). Identification of G-quadruplex clusters by high-throughput sequencing of whole-genome amplified products with a G-quadruplex ligand. Sci Rep.

[78] Rigo R, Palumbo M, Sissi C (2017). G-quadruplexes in human promoters: a challenge for therapeutic applications. Biochim Biophys Acta Gen Subj.

[79] Halder R, Halder K, Sharma P, Garg G, Sengupta S, Chowdhury S (2010). Guanine quadruplex DNA structure restricts methylation of CpG dinucleotides genome-wide. Mol Biosyst.

[80] Cree SL, Fredericks R, Miller A, Pearce FG, Filichev V, Fee C (2016). DNA G-quadruplexes show strong interaction with DNA methyltransferases in vitro. FEBS Lett.

[81] Mao SQ, Ghanbarian AT, Spiegel J, Martinez Cuesta S, Beraldi D, Di Antonio M (2018). DNA G-quadruplex structures mold the DNA methylome. Nat Struct Mol Biol.

[82] Yang C, Hu Y, Zhou B, Bao Y, Li Z, Gong C (2020). The role of m(6)A modification in physiology and disease. Cell Death Dis.

[83] Jara-Espejo M, Fleming AM, Burrows CJ (2020). Potential G-quadruplex forming sequences and N(6)-methyladenosine colocalize at human pre-mRNA intron splice sites. ACS Chem Biol.

[84] Fleming AM, Nguyen NLB, Burrows CJ (2019). Colocalization of m(6)A and G-quadruplex-forming sequences in viral RNA (HIV, Zika, Hepatitis B, and SV40) suggests topological control of adenosine N (6)-methylation. ACS Cent Sci.

[85] Iwasaki Y, Ookuro Y, Iida K, Nagasawa K, Yoshida W (2022). Destabilization of DNA and RNA G-quadruplex structures formed by GGA repeat due to N(6)-methyladenine modification. Biochem Biophys Res Commun.

[86] Horiuchi K, Kawamura T, Hamakubo T (2021). Wilms’ tumor 1-associating protein complex regulates alternative splicing and polyadenylation at potential G-quadruplex-forming splice site sequences. J Biol Chem.

[87] Yoshida A, Oyoshi T, Suda A, Futaki S, Imanishi M (2022). Recognition of G-quadruplex RNA by a crucial RNA methyltransferase component, METTL14. Nucleic Acids Res.

[88] Thorpe GW, Reodica M, Davies MJ, Heeren G, Jarolim S, Pillay B (2013). Superoxide radicals have a protective role during H2O2 stress. Mol Biol Cell.

[89] Fleming AM, Burrows CJ (2020). Iron Fenton oxidation of 2’-deoxyguanosine in physiological bicarbonate buffer yields products consistent with the reactive oxygen species carbonate radical anion not the hydroxyl radical. Chem Commun (Camb).

[90] Fleming AM, Burrows CJ (2020). Interplay of guanine oxidation and G-quadruplex folding in gene promoters. J Am Chem Soc.

[91] Vorlickova M, Tomasko M, Sagi AJ, Bednarova K, Sagi J (2012). 8-oxoguanine in a quadruplex of the human telomere DNA sequence. FEBS J.

[92] Neeley W, Essigmann J. Mechanisms of formation, genotoxicity, and mutation of guanine oxidation products. Chem Res Toxicol. 2006;19**:**491–505. 10.1021/tx0600043 16608160

[93] Singh Kukreti, Saso Kukreti (2019). Oxidative stress: role and response of short guanine tracts at genomic locations. Int J Mol Sci.

[94] Gros J, Rosu F, Amrane S, De Cian A, Gabelica V, Lacroix L (2007). Guanines are a quartet’s best friend: impact of base substitutions on the kinetics and stability of tetramolecular quadruplexes. Nucleic Acids Res.

[95] Fleming AM, Zhou J, Wallace SS, Burrows CJ (2015). A role for the fifth G-track in G-quadruplex forming oncogene promoter sequences during oxidative stress: do these “spare tires” have an evolved function?. ACS Cent Sci.

[96] Hognon C, Gebus A, Barone G, Monari A (2019). Human DNA telomeres in presence of oxidative lesions: the crucial role of electrostatic interactions on the stability of guanine quadruplexes. Antioxid (Basel).

[97] Bielskute S, Plavec J, Podbevsek P (2021). Oxidative lesions modulate G-quadruplex stability and structure in the human BCL2 promoter. Nucleic Acids Res.

[98] Tan J, Wang X, Phoon L, Yang H, Lan L (2020). Resolution of ROS-induced G-quadruplexes and R-loops at transcriptionally active sites is dependent on BLM helicase. FEBS Lett.

[99] Sanz LA, Hartono SR, Lim YW, Steyaert S, Rajpurkar A, Ginno PA (2016). Prevalent, dynamic, and conserved R-loop structures associate with specific epigenomic signatures in mammals. Mol cell.

[100] Teng Y, Yadav T, Duan M, Tan J, Xiang Y, Gao B (2018). ROS-induced R loops trigger a transcription-coupled but BRCA1/2-independent homologous recombination pathway through CSB. Nat Commun.

[101] Ghosh A, Rossi ML, Aulds J, Croteau D, Bohr VA (2009). Telomeric D-loops containing 8-oxo-2’-deoxyguanosine are preferred substrates for Werner and Bloom syndrome helicases and are bound by POT1. J Biol Chem.

[102] De Bont R, van Larebeke N (2004). Endogenous DNA damage in humans: a review of quantitative data. Mutagenesis.

[103] Banyasz A, Martinez-Fernandez L, Balty C, Perron M, Douki T, Improta R (2017). Absorption of low-energy UV radiation by human telomere G-quadruplexes generates long-lived guanine radical cations. J Am Chem Soc.

[104] Balanikas E, Banyasz A, Douki T, Baldacchino G, Markovitsi D (2020). Guanine radicals induced in DNA by low-energy photoionization. Acc Chem Res.

[105] Chen J, Jin X, Shen Z, Mei Y, Zhu J, Zhang X (2021). H2O2 enhances the anticancer activity of TMPyP4 by ROS-mediated mitochondrial dysfunction and DNA damage. Med Oncol.

[106] Le VH, Nagesh N, Lewis EA (2013). Bcl-2 promoter sequence G-quadruplex interactions with three planar and non-planar cationic porphyrins: TMPyP4, TMPyP3, and TMPyP2. PLoS ONE.

[107] O’Sullivan RJ, Karlseder J (2010). Telomeres: protecting chromosomes against genome instability. Nat Rev Mol Cell Biol.

[108] Srinivas N, Rachakonda S, Kumar R (2020). Telomeres and telomere length: a general overview. Cancers (Basel).

[109] Shay JW, Wright WE (2011). Role of telomeres and telomerase in cancer. Semin Cancer Biol.

[110] Sanders JL, Newman AB (2013). Telomere length in epidemiology: a biomarker of aging, age-related disease, both, or neither?. Epidemiol Rev.

[111] Reichert S, Stier A (2017). Does oxidative stress shorten telomeres in vivo? A review. Biol Lett.

[112] Cassidy A, De Vivo I, Liu Y, Han J, Prescott J, Hunter DJ (2010). Associations between diet, lifestyle factors, and telomere length in women. Am J Clin Nutr.

[113] Zglinicki TV (2002). Oxidative stress shortens telomeres. TRENDS Biochemical Sci.

[114] Shinji Oikawa SK (1999). Site-specific DNA damage at GGG sequence by oxidative stress may accelerate telomere shortening. FEBS Lett.

[115] von Zglinicki T (2002). Oxidative stress shortens telomeres. Trends Biochem Sci.

[116] Palm W, de Lange T (2008). How shelterin protects mammalian telomeres. Annu Rev Genet.

[117] Srinivas US, Tan BWQ, Vellayappan BA, Jeyasekharan AD (2019). ROS and the DNA damage response in cancer. Redox Biol.

[118] Lopez-Diazguerrero NE, Perez-Figueroa GE, Martinez-Garduno CM, Alarcon-Aguilar A, Luna-Lopez A, Gutierrez-Ruiz MC (2012). Telomerase activity in response to mild oxidative stress. Cell Biol Int.

[119] Ambrus A, Chen D, Dai J, Bialis T, Jones RA, Yang D (2006). Human telomeric sequence forms a hybrid-type intramolecular G-quadruplex structure with mixed parallel/antiparallel strands in potassium solution. Nucleic Acids Res.

[120] Lee H, Bose A, Lee C, Opresko P, Myong S. Molecular mechanisms by which oxidative DNA damage promotes telomerase activity. Nucleic Acids Res. 2017;45:11752–65. 10.1093/nar/gkx789 PMC5714237 28981887

[121] Bielskute S, Plavec J, Podbevsek P (2019). Impact of oxidative lesions on the human telomeric G-quadruplex. J Am Chem Soc.

[122] Frenkel K, Goldstein M, Teebor GJB. Identification of the cis-thymine glycol moiety in chemically oxidized and gamma-irradiated deoxyribonucleic acid by high-pressure liquid chromatography analysis. Biochemistry. 1981;20**:**7566–71. 10.1021/bi00529a035 7326245

[123] Fouquerel E, Parikh D, Opresko P (2016). DNA damage processing at telomeres: the ends justify the means. DNA Repair (Amst).

[124] Zhou J, Liu M, Fleming AM, Burrows CJ, Wallace SS (2013). Neil3 and NEIL1 DNA glycosylases remove oxidative damages from quadruplex DNA and exhibit preferences for lesions in the telomeric sequence context. J Biol Chem.

[125] Fleming AM, Burrows CJ (2017). 8-Oxo-7,8-dihydroguanine, friend and foe: Epigenetic-like regulator versus initiator of mutagenesis. DNA Repair.

[126] Fleming AM, Ding Y, Burrows CJ (2017). Oxidative DNA damage is epigenetic by regulating gene transcription via base excision repair. Proc Natl Acad Sci USA.

[127] Redstone SCJ, Fleming AM, Burrows CJ (2019). Oxidative modification of the potential G-quadruplex sequence in the PCNA gene promoter can turn on transcription. Chem Res Toxicol.

[128] Seifermann M, Epe B. Oxidatively generated base modifications in DNA: not only carcinogenic risk factor but also regulatory mark? Free Radic Biol Med. 2017;107:258–65. 10.1016/j.freeradbiomed.2016.11.018 27871818

[129] Fleming AM, Zhu J, Ding Y, Burrows CJ (2019). Location dependence of the transcriptional response of a potential G-quadruplex in gene promoters under oxidative stress. Nucleic Acids Res.

[130] Fedeles BI (2017). G-quadruplex-forming promoter sequences enable transcriptional activation in response to oxidative stress. Proc Natl Acad Sci USA.

[131] Fleming AM, Zhu J, Howpay Manage SA, Burrows CJ (2019). Human NEIL3 gene expression regulated by epigenetic-like oxidative DNA modification. J Am Chem Soc.

[132] Cogoi S, Ferino A, Miglietta G, Pedersen EB, Xodo LE (2018). The regulatory G4 motif of the Kirsten ras (KRAS) gene is sensitive to guanine oxidation: implications on transcription. Nucleic Acids Res.

[133] Cinque G, Ferino A, Pedersen EB, Xodo LE (2020). Role of poly [ADP-ribose] polymerase 1 in activating the Kirsten ras (KRAS) gene in response to oxidative stress. Int J Mol Sci.

[134] Edwards AD, Marecki JC, Byrd AK, Gao J, Raney KD (2021). G-Quadruplex loops regulate PARP-1 enzymatic activation. Nucleic Acids Res.

[135] Emara M, Fujimura K, Sciaranghella D, Ivanova V, Ivanov P, Anderson PJB, et al. Hydrogen peroxide induces stress granule formation independent of eIF2α phosphorylation. Biochem Biophys Res Commun. 2012;423:763–9. 10.1016/j.bbrc.2012.06.033 PMC3399031 22705549

[136] Arimoto-Matsuzaki K, Saito H, Takekawa M (2016). TIA1 oxidation inhibits stress granule assembly and sensitizes cells to stress-induced apoptosis. Nat Commun.

[137] Takahashi M, Higuchi M, Matsuki H, Yoshita M, Ohsawa T, Oie M (2013). Stress granules inhibit apoptosis by reducing reactive oxygen species production. Mol Cell Biol.

[138] Kedersha N, Ivanov P, Anderson P (2013). Stress granules and cell signaling: more than just a passing phase?. Trends Biochem Sci.

[139] Lyons SM, Achorn C, Kedersha NL, Anderson PJ, Ivanov P (2016). YB-1 regulates tiRNA-induced Stress Granule formation but not translational repression. Nucleic Acids Res.

[140] Somasekharan SP, El-Naggar A, Leprivier G, Cheng H, Hajee S, Grunewald TG (2015). YB-1 regulates stress granule formation and tumor progression by translationally activating G3BP1. J Cell Biol.

[141] Ivanov P, O’Day E, Emara MM, Wagner G, Lieberman J, Anderson P (2014). G-quadruplex structures contribute to the neuroprotective effects of angiogenin-induced tRNA fragments. Proc Natl Acad Sci USA.

[142] Ivanov P, Emara MM, Villen J, Gygi SP, Anderson P (2011). Angiogenin-induced tRNA fragments inhibit translation initiation. Mol cell.

[143] Lyons SM, Kharel P, Akiyama Y, Ojha S, Dave D, Tsvetkov V (2020). eIF4G has intrinsic G-quadruplex binding activity that is required for tiRNA function. Nucleic Acids Res.

[144] Li YR, King OD, Shorter J, Gitler AD (2013). Stress granules as crucibles of ALS pathogenesis. J Cell Biol.

[145] Ramaswami M, Taylor JP, Parker R (2013). Altered ribostasis: RNA-protein granules in degenerative disorders. Cell.

[146] Anderson P, Kedersha N, Ivanov P (2015). Stress granules, P-bodies and cancer. Biophys Acta.

[147] Adjibade P, St-Sauveur VG, Quevillon Huberdeau M, Fournier MJ, Savard A, Coudert L (2015). Sorafenib, a multikinase inhibitor, induces formation of stress granules in hepatocarcinoma cells. Oncotarget..

[148] Masuda Y, Vaziri ND, Li S, Le A, Hajighasemi-Ossareh M, Robles L (2015). The effect of Nrf2 pathway activation on human pancreatic islet cells. PLoS ONE.

[149] Fu J, Zheng H, Wang H, Yang B, Zhao R, Lu C (2015). Protective role of nuclear factor E2-related factor 2 against acute oxidative stress-induced pancreatic beta-cell damage. Oxid Med Cell Longev.

[150] Lee JM, Johnson JA (2004). An important role of Nrf2-ARE pathway in the cellular defense mechanism. J Biochem Mol Biol.

[151] Hayes JD, Dinkova-Kostova AT (2014). The Nrf2 regulatory network provides an interface between redox and intermediary metabolism. Trends Biochem Sci.

[152] Waller ZA, Howell LA, Macdonald CJ, O’Connell MA, Searcey M (2014). Identification and characterisation of a G-quadruplex forming sequence in the promoter region of nuclear factor (erythroid-derived 2)-like 2 (Nrf2). Biochem Biophys Res Commun.

[153] Dumas L, Herviou P, Dassi E, Cammas A, Millevoi S (2021). G-quadruplexes in RNA biology: recent advances and future directions. Trends Biochem Sci.

[154] Leppek K, Das R, Barna M (2018). Functional 5’ UTR mRNA structures in eukaryotic translation regulation and how to find them. Nat Rev Mol Cell Biol.

[155] Lee SC, Zhang J, Strom J, Yang D, Dinh TN, Kappeler K (2017). G-quadruplex in the NRF2 mRNA 5’ untranslated region regulates De Novo NRF2 protein translation under oxidative stress. Mol Cell Biol.

[156] Bryan HK, Olayanju A, Goldring CE, Park BK (2013). The Nrf2 cell defence pathway: Keap1-dependent and -independent mechanisms of regulation. Biochem Pharm.

[157] Tang YC, Hsiao JR, Jiang SS, Chang JY, Chu PY, Liu KJ (2021). c-MYC-directed NRF2 drives malignant progression of head and neck cancer via glucose-6-phosphate dehydrogenase and transketolase activation. Theranostics..

[158] Chen HH, Chang HH, Chang JY, Tang YC, Cheng YC, Lin LM (2017). Enhanced B-Raf-mediated NRF2 gene transcription and HATs-mediated NRF2 protein acetylation contributes to ABCC1-mediated chemoresistance and glutathione-mediated survival in acquired topoisomerase II poison-resistant cancer cells. Free Radic Biol Med.

[159] Naguib S, Backstrom JR, Gil M, Calkins DJ, Rex TS (2021). Retinal oxidative stress activates the NRF2/ARE pathway: An early endogenous protective response to ocular hypertension. Redox Biol.

[160] Zhang L, Yan T, Wang W, Wu Q, Li G, Li D (2021). AKT1 is positively regulated by G-quadruplexes in its promoter and 3’-UTR. Biochem Biophys Res Commun.

[161] Singh K, Lin J, Lecomte N, Mohan P, Gokce A, Sanghvi VR (2021). Targeting eIF4A-dependent translation of KRAS signaling molecules. Cancer Res.

[162] Moloney JN, Cotter TG (2018). ROS signalling in the biology of cancer. Semin Cell Dev Biol.

[163] Halliwell B (2007). Oxidative stress and cancer: have we moved forward?. Biochem J.

[164] Frenkel KJP (1992). therapeutics Carcinog-mediated Oxid formation Oxid DNA damage.

[165] Balasubramanian S, Hurley L, Neidle S. Targeting G-quadruplexes in gene promoters: a novel anticancer strategy? Nat Rev Drug Discov. 2011;10:261–75. 10.1038/nrd3428 PMC3119469 21455236

[166] Chaires J, Trent J, Gray R, Dean W, Buscaglia R, Thomas S, et al. An improved model for the hTERT promoter quadruplex. PLoS ONE. 2014;9:e115580. 10.1371/journal.pone.0115580 PMC4272262 25526084

[167] Feng Y, Yang D, Chen H, Cheng W, Wang L, Sun H (2016). Stabilization of G-quadruplex DNA and inhibition of Bcl-2 expression by a pyridostatin analog. Bioorg Med Chem Lett.

[168] Guo K, Gokhale V, Hurley LH, Sun D (2008). Intramolecularly folded G-quadruplex and i-motif structures in the proximal promoter of the vascular endothelial growth factor gene. Nucleic Acids Res.

[169] Talks K, Turley H, Gatter K, Maxwell P, Pugh C, Ratcliffe P, et al. The expression and distribution of the hypoxia-inducible factors HIF-1alpha and HIF-2alpha in normal human tissues, cancers, and tumor-associated macrophages. Am J Pathol. 2000;157:411–21. 10.1016/s0002-9440(10)64554-3 PMC1850121 10934146

[170] Chen H, Long H, Cui X, Zhou J, Xu M, Yuan G. Exploring the formation and recognition of an important G-quadruplex in a HIF1α promoter and its transcriptional inhibition by a benzo[c]phenanthridine derivative. J Am Chem Soc. 2014;136:2583–91. 10.1021/ja412128w 24450937

[171] Lu L, Wang M, Mao Z, Kang TS, Chen XP, Lu JJ (2016). A novel dinuclear iridium(III) complex as a G-quadruplex-selective probe for the luminescent switch-on detection of transcription factor HIF-1alpha. Sci Rep..

[172] Chen H, Long H, Cui X, Zhou J, Xu M, Yuan G (2014). Exploring the formation and recognition of an important G-quadruplex in a HIF1alpha promoter and its transcriptional inhibition by a benzo[c]phenanthridine derivative. J Am Chem Soc.

[173] Agostinis P, Berg K, Cengel KA, Foster TH, Girotti AW, Gollnick SO (2011). Photodynamic therapy of cancer: an update. CA Cancer J Clin.

[174] Sun Y, Hou YJ, Zhou QX, Chen JR, Zhang BW, Wang XS (2011). A new Co(III)-hypocrellin B complex with enhanced photonuclease activity. J Inorg Biochem.

[175] Falk-Mahapatra R, Gollnick SO (2020). Photodynamic therapy and immunity: an update. Photochem Photobiol.

[176] Shi T, Wang M, Li H, Wang M, Luo X, Huang Y (2018). Simultaneous monitoring of cell-surface receptor and tumor-targeted photodynamic therapy via TdT-initiated poly-G-quadruplexes. Sci Rep.

[177] Caterino M, D’Aria F, Kustov AV, Belykh DV, Khudyaeva IS, Starseva OM (2020). Selective binding of a bioactive porphyrin-based photosensitizer to the G-quadruplex from the KRAS oncogene promoter. Int J Biol Macromol.

[178] Kumari S, Bugaut A, Huppert JL, Balasubramanian S (2007). An RNA G-quadruplex in the 5’ UTR of the NRAS proto-oncogene modulates translation. Nat Chem Biol.

[179] Zhu L-N, Shi S, Yang L, Zhang M, Liu K-K, Zhang L-N (2016). Water soluble cationic porphyrin TMPipEOPP-induced G-quadruplex and double-stranded DNA photocleavage and cell phototoxicity. RSC Adv.

[180] Beniaminov AD, Novikov RA, Mamaeva OK, Mitkevich VA, Smirnov IP, Livshits MA (2016). Light-induced oxidation of the telomeric G4 DNA in complex with Zn(II) tetracarboxymethyl porphyrin. Nucleic Acids Res.

[181] Kawauchi K, Sugimoto W, Yasui T, Murata K, Itoh K, Takagi K (2018). An anionic phthalocyanine decreases NRAS expression by breaking down its RNA G-quadruplex. Nat Commun.

[182] Ferino A, Nicoletto G, D’Este F, Zorzet S, Lago S, Richter SN (2020). Photodynamic therapy for ras-driven cancers: targeting G-quadruplex RNA structures with bifunctional alkyl-modified porphyrins. J Med Chem.

[183] Haeusler AR, Donnelly CJ, Periz G, Simko EA, Shaw PG, Kim MS (2014). C9orf72 nucleotide repeat structures initiate molecular cascades of disease. Nature..

[184] Farhath MM, Thompson M, Ray S, Sewell A, Balci H, Basu S (2015). G-quadruplex-enabling sequence within the human tyrosine hydroxylase promoter differentially regulates transcription. Biochemistry..

[185] Wang M, Cai E, Fujiwara N, Fones L, Brown E, Yanagawa Y (2017). Odorant sensory input modulates DNA secondary structure formation and heterogeneous ribonucleoprotein recruitment on the tyrosine hydroxylase and glutamic acid decarboxylase 1 promoters in the olfactory bulb. J Neurosci.

[186] Lyu K, Chen SB, Chan CY, Tan JH, Kwok CK (2019). Structural analysis and cellular visualization of APP RNA G-quadruplex. Chem Sci.

[187] Dai J, Liu ZQ, Wang XQ, Lin J, Yao PF, Huang SL (2015). Discovery of small molecules for up-regulating the translation of antiamyloidogenic secretase, a disintegrin and metalloproteinase 10 (ADAM10), by binding to the G-quadruplex-forming sequence in the 5’ untranslated region (UTR) of its mRNA. J Med Chem.

[188] Koukouraki P, Doxakis E (2016). Constitutive translation of human alpha-synuclein is mediated by the 5’-untranslated region. Open Biol.

[189] Singh A, Kukreti R, Saso L, Kukreti S (2019). Oxidative stress: a key modulator in neurodegenerative diseases. Molecules.

[190] Mariani E, Polidori MC, Cherubini A, Mecocci P (2005). Oxidative stress in brain aging, neurodegenerative and vascular diseases: an overview. J Chromatogr B: Anal Technol Biomed Life Sci.

[191] Grigg JC, Shumayrikh N, Sen D (2014). G-quadruplex structures formed by expanded hexanucleotide repeat RNA and DNA from the neurodegenerative disease-linked C9orf72 gene efficiently sequester and activate heme. PLoS One.

[192] Stefan L, Denat F, Monchaud D. Deciphering the DNAzyme activity of multimeric quadruplexes: insights into their actual role in the telomerase activity evaluation assay. 2011;133:20405–15. 10.1021/ja208145d 22050329

[193] Gray LT, Puig Lombardi E, Verga D, Nicolas A, Teulade-Fichou MP, Londono-Vallejo A (2019). G-quadruplexes sequester free heme in living cells. Cell Chem Biol.

[194] Masud T, Soong C, Xu H, Biele J, Bjornson S, McKinney S (2021). Ubiquitin-mediated DNA damage response is synthetic lethal with G-quadruplex stabilizer CX-5461. Sci Rep.

[195] D’Aria F, Pagano B, Petraccone L, Giancola C (2021). KRAS promoter G-quadruplexes from sequences of different length: a physicochemical study. Int J Mol Sci.

[196] Angelika M, Burger FD, Christoph M, Schultes (2005). Tumor growth, consistent with telomere targeting and the G-quadruplex-interactive molecule BRACO-19 inhibits interference with telomerase function. Cancer Res.

[197] Gunaratnam M, Green C, Moreira JB, Moorhouse AD, Kelland LR, Moses JE (2009). G-quadruplex compounds and cis-platin act synergistically to inhibit cancer cell growth in vitro and in vivo. Biochem Pharm.

[198] Lagah S, Tan IL, Radhakrishnan P, Hirst RA, Ward JH, O’Callaghan C (2014). RHPS4 G-quadruplex ligand induces anti-proliferative effects in brain tumor cells. PLoS One.

[199] Salvati E, Leonetti C, Rizzo A, Scarsella M, Mottolese M, Galati R (2007). Telomere damage induced by the G-quadruplex ligand RHPS4 has an antitumor effect. J Clin Investig.

[200] Amato R, Valenzuela M, Berardinelli F, Salvati E, Maresca C, Leone S (2020). G-quadruplex stabilization fuels the ALT pathway in ALT-positive osteosarcoma cells. Genes (Basel).

[201] Teulade-Fichou MP, Carrasco C, Guittat L, Bailly C, Alberti P, Mergny JL (2003). Selective recognition of G-qQuadruplex telomeric DNA by a bis(quinacridine) macrocycle. J Am Chem Soc.

[202] Marie-Paule TF, Carolina C, Lionel G, Christian B, Patrizia A, Jean-Louis M (2003). Selective recognition of G-quadruplex telomeric DNA by a bis(quinacridine) macrocycle. JACS.

[203] Nagesh N, Sharma VK, Ganesh Kumar A, Lewis EA (2010). Effect of ionic strength on porphyrin drugs interaction with quadruplex DNA formed by the promoter region of C-myc and Bcl2 oncogenes. J Nucleic Acids.

[204] Qin Y, Rezler EM, Gokhale V, Sun D, Hurley LH (2007). Characterization of the G-quadruplexes in the duplex nuclease hypersensitive element of the PDGF-A promoter and modulation of PDGF-A promoter activity by TMPyP4. Nucleic Acids Res.

[205] Sun D, Liu WJ, Guo K, Rusche JJ, Ebbinghaus S, Gokhale V (2008). The proximal promoter region of the human vascular endothelial growth factor gene has a G-quadruplex structure that can be targeted by G-quadruplex-interactive agents. Mol Cancer Ther.

[206] Tang J, Wu J, Zhu R, Wang Z, Zhao C, Tang P (2021). Reversible photo-regulation on the folding/unfolding of telomere G-quadruplexes with solid-state nanopores. Analyst..

[207] Yan C, Xu J, Yao B, Yang L, Yao L, Liu G (2022). Facile design of multifunction-integrated linear oligonucleotide probe with multiplex amplification effect for label-free and highly sensitive GMO biosensing. Talanta.

[208] Muturi E, Meng F, Liu H, Jiang M, Wei H, Yang H (2021). Comprehensive analysis of G-quadruplexes in African swine fever virus genome reveals potential antiviral targets by G-quadruplex stabilizers. Front Microbiol.

[209] Cui X, Lin S, Yuan G (2012). Spectroscopic probing of recognition of the G-quadruplex in c-kit promoter by small-molecule natural products. Int J Biol Macromol.

[210] Rocca R, Moraca F, Costa G, Alcaro S, Distinto S, Maccioni E (2014). Structure-based virtual screening of novel natural alkaloid derivatives as potential binders of h-telo and c-myc DNA G-quadruplex conformations. Molecules..

[211] Srinivasan S, Ranganathan V, DeRosa MC, Murari BM (2019). Comparison of turn-on and ratiometric fluorescent G-quadruplex aptasensor approaches for the detection of ATP. Anal Bioanal Chem.

[212] Wen LN, Xie MX (2017). Spectroscopic investigation of the interaction between G-quadruplex of KRAS promoter sequence and three isoquinoline alkaloids. Spectrochim Acta A: Mol Biomol Spectrosc.

[213] Gu HP, Lin S, Xu M, Yu HY, Du XJ, Zhang YY (2012). Up-regulating relaxin expression by G-quadruplex interactive ligand to achieve antifibrotic action. Endocrinology..

[214] Endoh T, Sugimoto N (2013). Unusual -1 ribosomal frameshift caused by stable RNA G-quadruplex in open reading frame. Anal Chem.

[215] Shan C, Yan JW, Wang YQ, Che T, Huang ZL, Chen AC (2017). Design, synthesis, and evaluation of isaindigotone derivatives to downregulate c-myc transcription via disrupting the interaction of NM23-H2 with G-quadruplex. J Med Chem.

[216] Yan JW, Chen SB, Liu HY, Ye WJ, Ou TM, Tan JH (2014). Development of a new colorimetric and red-emitting fluorescent dual probe for G-quadruplex nucleic acids. Chem Commun (Camb).

[217] Wang SK, Wu Y, Wang XQ, Kuang GT, Zhang Q, Lin SL (2017). Discovery of small molecules for repressing cap-independent translation of human vascular endothelial growth factor (hVEGF) as novel antitumor agents. J Med Chem.

[218] Guillon J, Cohen A, Boudot C, Valle A, Milano V, Das RN, et al. Design, synthesis, and antiprotozoal evaluation of new 2,4-bis[(substitutedaminomethyl)phenyl]quinoline, 1,3bis[(substituted-aminomethyl)phenyl]isoquinoline and 2,4-bis[(substitutedaminomethyl)phenyl]quinazoline derivatives. J Enzyme Inhib Med Chem. 2020;35:432–59. 10.1080/14756366.2019.1706502 PMC6968685 31899980

[219] Tawani A, Mishra SK, Kumar A (2017). Structural insight for the recognition of G-quadruplex structure at human c-myc promoter sequence by flavonoid Quercetin. Sci Rep.

[220] Tawani A, Kumar A (2015). Structural Insight into the interaction of flavonoids with human telomeric sequence. Sci Rep.

[221] Laguerre A, Hukezalie K, Winckler P, Katranji F, Chanteloup G, Pirrotta M (2015). Visualization of RNA-quadruplexes in live cells. J Am Chem Soc.

[222] Weldon C, Dacanay JG, Gokhale V, Boddupally PVL, Behm-Ansmant I, Burley GA (2018). Specific G-quadruplex ligands modulate the alternative splicing of Bcl-X. Nucleic Acids Res.

[223] Montoya JJ, Turnidge MA, Wai DH, Patel AR, Lee DW, Gokhale V (2019). In vitro activity of a G-quadruplex-stabilizing small molecule that synergizes with Navitoclax to induce cytotoxicity in acute myeloid leukemia cells. BMC Cancer.

[224] Kumarasamy VM, Sun D (2017). Demonstration of a potent RET transcriptional inhibitor for the treatment of medullary thyroid carcinoma based on an ellipticine derivative. Int J Oncol.

[225] Hu MH, Wu TY, Huang Q, Jin G (2019). New substituted quinoxalines inhibit triple-negative breast cancer by specifically downregulating the c-MYC transcription. Nucleic Acids Res.

[226] Calabrese DR, Chen X, Leon EC, Gaikwad SM, Phyo Z, Hewitt WM (2018). Chemical and structural studies provide a mechanistic basis for recognition of the MYC G-quadruplex. Nat Commun.

[227] Yao YX, Xu BH, Zhang Y (2018). CX-3543 promotes cell apoptosis through downregulation of CCAT1 in colon cancer cells. Biomed Res Int.

[228] Xu H, Di Antonio M, McKinney S, Mathew V, Ho B, O’Neil NJ (2017). CX-5461 is a DNA G-quadruplex stabilizer with selective lethality in BRCA1/2 deficient tumours. Nat Commun.

[229] Sullivan HJ, Chen B, Wu C (2020). Molecular dynamics study on the binding of an anticancer DNA G-quadruplex stabilizer, CX-5461, to human telomeric, c-KIT1, and c-Myc G-quadruplexes and a DNA duplex. J Chem Inf Model.

[230] Zhu M, Wu W, Togashi Y, Liang W, Miyoshi Y, Ohta T (2021). HERC2 inactivation abrogates nucleolar localization of RecQ helicases BLM and WRN. Sci Rep.

[231] Gamelas SRD, Moura NMM, Habraken Y, Piette J, Neves M, Faustino MAF (2021). Tetracationic porphyrin derivatives against human breast cancer. J Photochem Photobiol B.

